# Alcohol Use, Stigmatizing/Discriminatory Attitudes, and HIV High-Risk Sexual Behaviors among Men Who Have Sex with Men in China

**DOI:** 10.1155/2014/143738

**Published:** 2014-03-27

**Authors:** Meizhen Liao, Dianmin Kang, Xiaorun Tao, Jennifer Huang Bouey, Muktar H. Aliyu, Yuesheng Qian, Guoyong Wang, Xiaoguang Sun, Bin Lin, Zhenqiang Bi, Yujiang Jia

**Affiliations:** ^1^Institution for HIV/AIDS Control and Prevention and Shandong Key Laboratory for Epidemic Disease Control and Prevention, Shandong Center for Disease Control and Prevention, Jinan, Shandong 250014, China; ^2^Department of International Health, Georgetown University, Washington, DC 20057, USA; ^3^Department of Preventive Medicine, Vanderbilt University School of Medicine, Nashville, TN 37232, USA; ^4^Vanderbilt Institute for Global Health, Vanderbilt University, Nashville, TN 37232, USA

## Abstract

*Objective*. This research was conducted to assess the correlates of alcohol consumption and HIV/AIDS-related stigmatizing and discriminatory attitudes among men who have sex with men (MSM) in Shandong province, China. *Methods*. A cross-sectional survey provided demographics, sexual behaviors, illicit drug use, alcohol consumptions, and service utilization. *Results*. Of 1,230 participants, 82.8% were single, 85.7% aged <35 years, 47.2% had college or higher education, and 11.7% drank alcohol >3 times per week in the past six months. The average total score of stigmatizing and discriminatory attitude was 37.4 ± 4.4. More frequent episodes of alcohol use were independently associated with higher levels of HIV/AIDS-related stigma and discrimination, unprotected anal sex, bisexual identity, multiple male sex partners, drug use, and lower levels of education. Expressing higher levels of HIV/AIDS-related stigmatizing and discriminatory attitudes was independently associated with alcohol use, unprotected male anal sex, bisexuals, more male sex partners, commercial sex with men, and non-receipt of peer education in the past year. *Conclusion*. HIV/AIDS-related stigmatizing and discriminatory attitudes are common and associated with alcohol use and unprotected sex among MSM. The finding highlights the needs to develop programs that would reduce HIV/AIDS-related stigmatizing and discriminatory attitudes and strengthen alcohol use prevention and risk reduction initiatives among MSM.

## 1. Introduction

The rapid rise in HIV prevalence among men who have sex with men (MSM) in many cities across the nation has drawn attention to the dynamics of the HIV epidemic in China [1–3]. It is estimated that about 2.2% of Chinese adult males had sex with another male [4]. According to the 2011 estimates for the HIV/AIDS epidemic in China, 81.6% of 48,000 HIV new cases were infected through sexual contact, 29.4% of those infected from sexual contact were through homosexual contact [2]. Under the strong influence of Confucianism and collectivism, Chinese traditional culture emphasizes familial responsibilities; MSM behaviors are highly stigmatized and MSM face strong social pressure to hide their identity [5]. On the other hand, stigma surrounding HIV/AIDS has been shown to act as a barrier to HIV prevention, treatment, and care [6, 7]. People who hold stigmatizing attitudes are also less likely to adopt preventive behaviors and more likely to have multiple sexual partners, a commercial sex partner, and some other HIV-related high-risk behaviors [8, 9].

Alcohol consumption has increased considerably in China in the past three decades, accompanying a rapidly expanding economy, urbanization, and globalization [10]. The global literature suggests that alcohol consumption is associated with a number of sexual risk behaviors and outcomes, including premarital intercourse, multiple sexual partners, and unprotected sex [11, 12]. As the most commonly used legal substance, drinking-related exposures to sexual risk behaviors and drinkers' increased biological susceptibility could lead to an increased risk of HIV and other sexually transmitted infections (STIs) [11]. Drink and HIV-related stigma/discrimination are common in the MSM community. Understanding the relationship between stigma, alcohol use, and sexual behavior among MSM has important intervention implications. The purpose of this study is to assess correlates for alcohol use and HIV/AIDS-related stigmatizing and discriminatory attitudes among MSM in Shandong province, China.

## 2. Methods

### 2.1. Study Participants

A cross-sectional study was conducted among MSM to collect demographic, sexual behaviors, illicit drug use, alcohol consumptions, and health service utilization by local Center for Disease Control and Prevention (CDC) in three cities, Jinan, Qingdao, and Yantai, in Shandong province, China, from April to June in 2011. Prior to the recruitment of the participants, we conducted informative research including in-depth interviews with key informants to gather the background information among MSM, the venues to access them, and selection of the candidates of the first group for interview. Participants were recruited from gay-oriented venues such as bars, night clubs, tea houses, bathhouses, saunas, public parks and bathrooms, outdoor cruising areas, and HIV testing sites. After these initial participants were approached and interviewed by trained public health staff, we asked the participants to refer their peers to attend the study. A mixed recruitment method, including community outreach, venue-based recruitment, and internet advertisement, was also applied in the study. All potential participants were invited for eligibility assessments. The enrollment criteria included male, 18 years of age or older, self-reported ever having sex with another male in the past 12 months, and willing to complete the study. Survey information was collected anonymously and handled in a confidential manner. Verbal informed consent was obtained from all participants before the interview. Voluntary participation, anonymity, and confidentiality were ensured for all participants. The study was approved by the Institutional Review Board of Shandong Center for Disease Control and Prevention.

### 2.2. Measures

Structured questionnaire-based interviews provided demographics (age, marital status, ethnicity, residency status and education, sexual, drug use, and alcohol drinking behaviors, access and utilization of HIV-related prevention services, and stigma and discriminatory attitudes towards People Living With HIV/AIDS (PLWHA)). The term “bisexual” was used for participants who were married or cohabiting with women or reported ever having sex with a woman in the past 6 months. Attitudes towards PLWHA were measured by asking participants about their agreement/disagreement (1 = “yes,” 2 = “no”) with 22 statements [13]. The scale was adapted from two pilot investigations tested in Thailand and Zimbabwe and measured 3 dimensions of HIV/AIDS-related stigma and discrimination: shame, blame, and social isolation; perceived discrimination; and equity. The reliability (alpha) of the stigma measure was 0.83. The scale included questions such as “People living with HIV/AIDS should be ashamed”; “People living with HIV/AIDS face neglect from their family”; “People living with HIV/AIDS do not deserve any support.” Items were summed to create total scale scores, with a range of 22–44 where a higher score means lower stigma and lower score indicates a higher stigma. Frequency of alcohol use was determined directly from responses to a single questionnaire item in which respondents were asked how often they drank in the past six months. Responses included “about every day,” “5-6 days a week,” “3-4 days a week,” “1-2 days a week,” “2-3 days a month,” “less often than monthly,” and “never.” In the final analysis, the categories “About every day,” “5-6 days a week,” and “3-4 days a week” were collapsed into a single variable “more than 3 days per week,” because of the low frequencies of individual responses when the original categories were considered separately. Serum samples were screened for HIV-1 antibodies by enzyme-linked immunosorbent assay (ELISA; Vironostika HIV Uni-Form plus O, bioMerieux, Holland) and confirmed by Western Blot test (HIV Blot 2.2 WB, Genelabs Diagnostics). Syphilis screening was performed by rapid plasma regain (RPR; Shanghai Rongsheng, China) and confirmed by Treponema pallidum particle assay test (TPPA; Fujirebioinc, Japan). Pre- and posttesting counseling were provided by local CDC.

### 2.3. Statistical Analysis

Survey data and blood testing results were recorded and assessed for congruency using EpiData software (EpiData 6.4 for Windows, the EpiData Association Odense, Denmark). The Statistical Program for Social Sciences software (SPSS software, Version 15.0; SPSS Inc., Chicago, IL, US) was utilized for all analyses. Univariate analyses were conducted for demographic, sexual, drug use, and drinking variables and related prevention services. Multivariable logistic regression analyses were conducted using a stepwise backward sequence. Variables with *P* < 0.05 in multivariable analysis were determined as statistically significant. Multiple linear regression analysis was applied to determine which predictors were independently associated with total stigma scale scores after controlling for potential confounders.

## 3. Results

### 3.1. Characteristics of Participants

Of 1,230 eligible participants, 82.8% were single, 85.7% were between 18 and 35 years of age, nearly half (47.2%) had college or higher levels of education, 28.6% were married or cohabiting with women or reported ever having sex with women in the past 6 months. More than two-thirds of respondents (68.7%) identified themselves as homosexual, a quarter (26.3%) identified themselves as bisexual, and 1.4% identified themselves as heterosexual; 19.4% were non-Shandong province residents, and 2.0% belonged to a non-Han minority ethnic group (Table 1).

### 3.2. Sexual Behaviors and Prevalence Rates of HIV and Syphilis

Approximately 91.4% of participants reported having had sex with men in the past six months, 54.3% had more than two male sex partners in the past week, 70.8% used a condom at last anal sex, and 31.3% consistently used condoms in the past 6 months with male partners. In addition, 27.2% of respondents admitted having had commercial sex with men, with only 29.8% reporting consistent condoms use in the past 6 months. Further, 21.5% of participants reported ever selling sex to another man, with only 31.1% of them reporting consistent condom use in the past 6 months. About one-quarter of respondents (23.4%) had sex with a female in the past 6 months, with one-third (32.5%) reporting consistent condom use over the same time period. Only 1.1% of participants reported ever using illicit drugs. Approximately half (50.7%) of participants received HIV testing in the past year, and three-quarters (75.2%) and 41.3% received condom promotion/HIV testing and counseling and peer education, respectively. Of all participants, 1.6% were HIV-infected and 6.8% were syphilis-infected (Table 2).

### 3.3. Correlates for Drinking Behaviors

Of the participants, 11.7% reported drinking alcohol ≥3 times per week in the past 6 months. Multivariable logistic regression analysis that indicated drinking behaviors was associated with higher levels of HIV/AIDS-related stigma and discrimination (AOR = 0.92, 95% CI: 0.87–0.96), unprotected male anal sex in the past 6 months (AOR = 1.9, 95% CI: 1.1–3.3), bisexual identity (AOR = 2.2, 95% CI: 1.3–3.9), more male sex partners in the past week (AOR = 1.5, 95% CI: 1.0–2.4; ≥2), drug use (AOR = 6.7, 95% CI: 2.0–22.3), and high school or lower education level (AOR = 1.6, 95% CI: 1.0–2.6) (Table 3).

### 3.4. Correlates for Stigma and Discrimination

**Figure 1:**
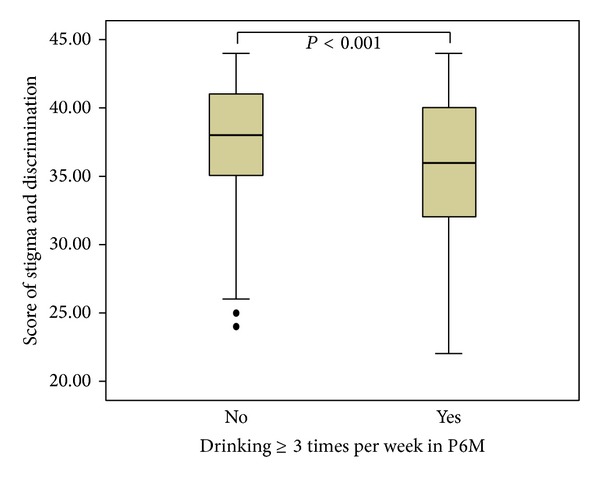
Comparisons of HIV/AIDS-related stigmatizing and discriminatory attitudes with the alcohol consumption for the participants who reported drinking ≥3 times per week in the past 6 months (P6M) in Shandong province, China.

The total score for stigmatizing and discriminatory attitudes among participants was 37.4 ± 4.4, ranging from 22 to 44. The multivariate linear regression model indicated that MSM who drank alcohol ≥3 times per week in the past 6 months (A*β* = 1.5, 95% CI: 0.8–2.3), had unprotected male anal sex in the past 6 months (A*β* = 1.7, 95% CI: 1.1–2.2), have bisexual identity (A*β* = 0.9, 95% CI: 0.4–1.4), had more male sex partners in past week (A*β* = 1.9, 95% CI: 1.3–2.4; ≥2), had commercial sex with man in the past 6 months (A*β* = 0.8, 95% CI: 0.2–1.4), and are non-receipt of peer education in the past year (A*β* = 1.7, 95% CI: 1.2–2.2) were more likely to have lower HIV/AIDS-related stigma/discrimination score and thus exhibit more negative attitudes (Table 3, Figure 1).

## 4. Discussion

To the best of our knowledge, this is the first study from China that assesses HIV/AIDS-related stigmatizing and discriminatory attitudes and its relationship with alcohol consumption among MSM. This study revealed that HIV/AIDS-related stigmatizing and discriminatory attitudes were common among MSM and associated with drinking behavior, unprotected anal sex, and commercial sex among MSM in Shandong province, China. Confronting a rapid rise of HIV incidence among MSM in China, common negative attitudes towards PLWHA, excessive alcohol use behaviors, and bisexual and unprotected sex among this group have become emerging challenges in containing the epidemic. Stigma is particularly relevant to prevention and treatment in the HIV/AIDS pandemic [14]. The negative attitudes towards persons living with HIV/AIDS (PLWHA) are often associated with self-imposed isolation that results from individuals' reluctance to access services due to fear that family and community members may shun them for their drinking and drug use behaviors [15, 16]. On the other hand, Chinese cultural norms encourage social drinking. Alcohol is commonly consumed in social settings, particularly by men, as a normal part of their social life, to identify with new friends, maintain good relations, and celebrate events among friends. Other researchers have shown a consistent association between alcohol use and several sexual risk behaviors and HIV/STIs across both high-risk groups and general populations in China [17, 18]. Hence, in confronting the rapid expansion of the HIV/AIDS epidemic among MSM in China, the findings of the present study underline the urgent need to reduce the stigmatizing and discriminatory attitudes towards PLWHA and call for the need for alcohol use prevention and risk reduction among MSM communities.

This study shows that self-identification as bisexual is independently associated with drinking behaviors and with more negative attitudes towards PLWHA. Shandong is the second most populous province in China and the home of Confucius. Under the strong influence of Confucianism and collectivism, MSM behaviors are stigmatized and MSM face strong social pressure [5, 19]. Such social environments may lead MSM to hide their sexual orientation by unwillingly engaging in heterosexual relationships [20]. Many of them continue to be married and maintain sexual relationships with their wives while maintaining concurrent but hidden homosexual relationships. Nearly one-third of MSM are married in China, and an even higher proportion reported having had sex with women [21, 22]. Our previous study showed approximately 40% of MSM being married or ever having had heterosexual behavior in Shandong province [23]. These bisexual and marital relationships have a strong impact on the HIV epidemic from MSM population to general population. Studies from different countries documented the high rate of unprotected sexual behaviors between spouses and/or regular heterosexual partners tends [22, 24, 25]. Therefore, bisexually active men in China could play a critical bridging role in spreading HIV and other STIs from their high-risk male sexual partners to low-risk female partners, for example, their wives [26, 27]. Under social pressure, bisexual MSM may further reinforce the negative attitudes towards PLWHA and worsen drinking behaviors.

While stigma is widely invoked as a major facilitator of the HIV epidemic [28], only few studies have demonstrated an association between stigma and increased risk behavior. This study showed that unprotected male anal sex in the past 6 months and ≥2 male sex partners in the past week are independently associated with drinking behavior and more negative attitudes towards PLWHA. Other studies that have demonstrated drinking behaviors will result in multiple sexual partners and unprotected sex [11, 12]. A cross-sectional study of MSM in 5 cities in Jiangsu province showed that heavy alcohol consumption was linked to unprotected anal intercourse (OR = 2.32) compared with nondrinkers or light drinkers [29]. Alcohol consumption may directly impair judgment and cause social disinhibition, resulting in an increased likelihood of unprotected sexual encounters. The other explanation involves a social environment model, in which an association occurs because the social environment of drinking overlaps with an environment that facilitates meeting potential casual sexual partners. Stigma can have significant adverse effects on health and disease transmission by promoting delays in seeking care and reluctance to follow medical advice [28]. One study among Chinese migrants found that persons holding stigmatizing beliefs were more likely to have multiple sexual partners, a commercial sex partner, and a STI [30]. The findings of the present study suggest that common multiple sex partners and existing sexual network among this group could offer an intervention opportunity to reduce HIV/AIDS-related stigmatizing and discriminatory attitudes and strengthen alcohol use prevention and risk reduction initiatives among MSM.

This study also found that MSM who ever received peer education in the past year were more likely to express lower negative attitudes towards PLWHA. Peer education could relieve their social pressure, persuade MSM to receive HIV counseling and testing, and facilitate early diagnosis, timely prevention, and linkage to care and medical treatment. Ti and Kerr showed that by creating peer-involved HIV testing clinics and pairing physicians with peers, high-risk groups may be more likely to use these services without fear of being discriminated by healthcare workers [31]. By shifting delivery of care from healthcare professionals to peers, or by incorporating peer workers into professionally led services, a reduction in stigma and discrimination in these settings may be achieved. The findings of the present study underline the effect of peer education and the need to deliver conventional HIV/AIDS services directly to MSM to reduce the stigmatization and discrimination among MSM communities.

These studies highlighted the importance of policy considerations for stigma, alcohol use, and its related sexual risk among MSM. This study is not without limitations. First, the stigma scale may provoke socially desirable answers from respondents. Participants may feel embarrassed to openly express stigmatizing and discriminatory attitudes towards PLWHA during face-to-face interviews. This study might therefore underestimate the true levels of negative attitudes and risk behavior. Second, the nature of the cross-sectional study design precluded identification of causal relationships. Third, since this study did not assess the cooccurrence of drinking behavior with inconsistent condom use or multiple sex partners, any inference on the causal relationship between these variables is impossible. Fourth, the variables used to characterize alcohol consumption are not a complete list and may miss some opportunities to capture all true relationships.

Despite its many limitations, this study provides important information for further research and suggests that MSM who experience stigmatizing and discriminatory attitudes towards PLWHA and exhibit patterns of excessive alcohol use may be at higher risk for increased numbers of sexual partners and for bisexual and unprotected sex. Common HIV/AIDS-related stigmatizing and discriminatory attitudes, drinking behaviors, bisexual identity, and unprotected sex among this group have become emerging challenges in containing the HIV epidemic. The findings of this study highlight the urgent need to develop programs that would target HIV/AIDS-related stigmatizing and discriminatory behavior and strengthen alcohol use prevention and risk reduction strategies among MSM, in the context of rapid social changes that are occurring in China.

## Supplementary Material

Table 1. Demographics and Biological Outcomes among Men Who Have Sex with Men in Shandong Province, China.
Table 2. Sex and Drug Use Behavior, Stigma and Discrimination, Alcohol Consumption, HIV Knowledge and HIV Prevention Services among Men Who Have Sex with Men in Shandong Province, China.
Table 3. Predictors for Stigma and Discrimination, Alcohol Consumption among Men Who Have Sex with Men, Shandong Province, China.
Figure 1. Comparisons of HIV/AIDS Related Stigmatizing and Discriminatory Attitudes with the Alcohol Consumption for the Participants Who Reported Ever Drinking≥3 Times per Week in Past 6 Months (P6M), Shandong Province, China.

## Figures and Tables

**Table 1:** Demographics and biological outcomes among men who have sex with men in Shandong province, China.

Variables	Total		Drinking (≥3 times/week)		Stigma and discrimination
	*N*	%	*N*	%	±SD
Total	1230		144	11.7	37.4 ± 4.4
*Demographics *					
Study sites					
Jinan	400	32.5	48	12.0	39.0 ± 3.8
Qingdao	400	32.5	55	13.7	33.8 ± 3.8^‡^
Yantai	430	35.0	41	9.5	39.4 ± 2.9
Recruited venue					
Bars, night clubs, or tea houses	329	26.7	49	14.9	36.7 ± 4.2
Bathhouses or sauna	161	13.1	24	14.9	33.8 ± 3.8^‡^
Outdoor cruising area	98	8.0	11	11.2	34.1 ± 3.9^‡^
Internet or HIV testing sites	642	52.2	60	9.3	39.2 ± 3.6
Age (years)					
<25	548	44.6	53	9.7	37.8 ± 4.2
25–34	505	41.1	50	9.9	37.4 ± 4.4
≥35	177	14.3	41	23.2^‡^	36.1 ± 4.6^‡^
Marital status					
Single/separated	1018	82.8	97	9.5	37.7 ± 4.2
Married or cohabitating	212	17.2	47	22.2^‡^	35.9 ± 4.6^‡^
Residency					
Shandong province	991	80.6	107	10.8	37.4 ± 4.4
Non-Shandong province	239	19.4	37	15.5	37.6 ± 4.2
Ethnicity group					
Han	1206	98.0	140	11.6	37.4 ± 4.4
Others	24	2.0	4	16.7	38.8 ± 3.5
Occupation					
Student	187	15.2	6	3.2	38.3 ± 4.3
Commercial service	539	43.8	80	14.8	37.7 ± 4.2
Farmer	117	9.5	8	6.8	38.9 ± 4.0
Full time employee	268	21.8	33	22.9^‡^	37.0 ± 4.3
Housework and/or unemployed	119	9.7	17	14.3	34.6 ± 4.3^‡^
Education					
High school or lower	649	52.8	94	14.5^†^	36.9 ± 4.5^‡^
College or higher	581	47.2	50	8.6	38.0 ± 4.1
Duration of residence in current location (years)					
≥2	815	66.3	97	11.9	37.4 ± 4.4
<2	415	33.7	47	11.3	37.4 ± 4.2
Self-identified sexual orientation					
Homosexual	845	68.7	73	8.6	37.6 ± 4.2
Heterosexual	17	1.4	6	35.3^‡^	34.6 ± 4.7^‡^
Bisexual	323	26.3	61	18.9	36.9 ± 4.6
Do not know	45	3.7	4	8.9	39.2 ± 3.1
Being married or cohabitating/ ever had sex with woman in past 6 months					
Homosexual	878	71.4	74	8.4	37.8 ± 4.3
Bisexual	352	28.6	70	19.9^‡^	36.6 ± 4.5^‡^
*Biological outcome *					
HIV status					
Negative	1208	98.4	142	11.8	37.4 ± 4.3
Positive	20	1.6	2	10.0	38.3 ± 4.4
Syphilis status					
Negative	1144	93.2	130	11.4	37.5 ± 4.4
Positive	84	6.8	14	16.7	36.4 ± 4.2*

Total *N* for each subgroup may not add up to the total due to missing data; P6M: in the past 6 months; *P < 0.05; ^†^ *P* < 0.01; ^‡^ *P* < 0.001; NA: not applicable.

**Table 2:** Sex and drug use behavior, stigma and discrimination, alcohol consumption, HIV knowledge, and HIV prevention services among men who have sex with men in Shandong province, China.

Variables	Total		Drinking (≥3 times/week)		Stigma and discrimination
	*N*	%	*N*	%	±SD
*Sexual and drug use behaviors *					
Age of first sex (years)					
≤20	661	53.7	70	10.6	38.0 ± 4.1
>20	569	46.3	74	13.0	36.7 ± 4.6^‡^
Sex with man in past 6 months					
No	105	8.6	6	5.7	39.4 ± 3.2
Yes	1123	91.4	138	12.3	37.2 ± 4.4^‡^
Number of male sex partners in the past week					
<2	499	45.7	43	8.6	38.8 ± 3.9
≥2	592	54.3	92	15.5^†^	35.9 ± 4.4^‡^
Condom use during sex with man in the last sex					
Yes	794	70.8	89	11.2	37.6 ± 4.3
No	327	29.2	48	14.7	36.4 ± 4.5^‡^
Condom use during sex with man in past 6 months					
Always	351	31.3	27	7.7	39.1 ± 3.4
Sometimes or never	770	68.7	111	14.4^†^	36.4 ± 4.5^‡^
Commercial sex with man in past 6 months					
Yes	306	27.2	51	16.7^†^	35.7 ± 4.2^‡^
No	818	72.8	86	10.5	37.8 ± 4.3
Condom use with paid male partner during the last sex					
No	84	6.8	18	21.4	34.3 ± 4.4^‡^
Yes	222	72.5	33	14.9	36.2 ± 4.0
Condom use with paid male sex partners in past 6 months					
Always	91	29.8	12	13.2	38.4 ± 3.4
Sometimes or never	214	70.2	38	17.8	34.5 ± 4.0^‡^
Sold sex to man in past 6 months					
Yes	264	21.5	43	16.3*	36.1 ± 4.2^‡^
No	966	78.5	101	10.5	37.8 ± 4.3
Condom use in the last time with male partner who sold sex					
No	56	21.4	9	16.1	34.8 ± 4.3^‡^
Yes	206	78.6	34	16.5	36.4 ± 4.1
Condom use in past 6 months with male partners who sold sex					
Always	82	31.1	12	14.6	38.8 ± 3.2
Sometimes or never	182	68.9	31	17.0	34.9 ± 4.1^‡^
Sex with woman in past 6 months					
Yes	287	23.4	61	21.3^‡^	36.8 ± 4.5^†^
No	942	76.6	83	8.8	37.6 ± 4.3
Condom use with female partners in the last sex act					
Yes	149	52.1	23	15.4	37.7 ± 4.1
No	137	47.9	37	27.0*	35.9 ± 4.7^‡^
Condom use with female partners in past 6 months					
Always	93	32.5	14	15.1	38.4 ± 3.7
Sometimes or never	193	67.5	46	23.8	36.1 ± 4.6^‡^
Drug use					
No	1210	98.9	138	11.4	37.4 ± 4.4
Yes	13	1.1	6	46.2^†^	36.9 ± 3.8
*HIV-related prevention services in the past year *					
Condom promotion/VCT					
Yes	925	75.2	116	12.5	38.1 ± 4.1
No	305	24.8	28	9.2	37.2 ± 4.4^†^
Received peer education					
Yes	508	41.3	69	13.6	38.7 ± 4.0
No	722	58.7	75	10.4	36.5 ± 4.3^‡^
Had free HIV test in the past year					
Yes	624	50.7	87	13.9*	38.5 ± 3.9
No	606	49.3	57	9.4	36.3 ± 4.5^‡^
*Drink ≥3 times per week in P6M *					
No	1086	88.3	—	—	37.6 ± 4.2
Yes	144	11.7	—	—	35.4 ± 5.2^‡^

**Table 3:** Predictors for stigma and discrimination, alcohol consumption among men who have sex with men in Shandong province, China.

Model 1 predictors for drink (≥3 times per week in P6M)	*N* (%)	OR (95% CI)	AOR (95% CI)
Higher level of stigma and discrimination (continuous)	35.4 ± 5.2	0.90 (0.86–0.93)^‡^	0.92 (0.87–0.96)^‡^
Unprotected male anal sex in P6M	111 (14.4)	2.0 (1.3–3.1)^†^	1.9 (1.1–3.3)*
Bisexual identity/orientation	70 (19.9)	2.7 (1.9–3.8)^‡^	2.2 (1.3–3.9)^†^
Number of male sex partners in the past week ≥2	92 (15.5)	2.0 (1.3–2.9)^†^	1.5 (1.0–2.4)*
Drug use	6 (46.2)	6.7 (2.2–20.1)^†^	6.7 (2.0–22.3)^†^
High school or lower education level	94 (14.5)	1.7 (1.1–2.5)^†^	1.6 (1.0–2.6)*

Model 2 predictors for stigma and discrimination	Mean ± SD	*β* (95% CI)	Adjusted *β* (95% CI)

Drink ≥3 times per week in P6M	35.4 ± 5.2	2.2 (1.5–3.0)^‡^	1.5 (0.8–2.3)^‡^
Unprotected male anal sex in P6M	36.4 ± 4.5	2.8 (2.2–3.3)^‡^	1.7 (1.1–2.2)^‡^
Bisexual identity/orientation	36.6 ± 4.5	1.2 (0.6–1.7)^‡^	0.9 (0.4–1.4)^†^
Number of male sex partners in past week ≥2	35.9 ± 4.4	2.9 (2.4–3.4)^‡^	1.9 (1.3–2.4)^‡^
Commercial sex with man in P6M	35.7 ± 4.2	2.1 (1.5–2.6)^‡^	0.8 (0.2–1.4)^†^
Never received peer education in the past year	36.5 ± 4.3	2.3 (1.8–2.7)^‡^	1.7 (1.2–2.2)^‡^

Multivariable logistic regression analysis was applied for alcohol consumption (Model 1); multivariable linear regression model was performed for stigma and discrimination (Model 2); P6M: in the past 6 months; OR: odds ratio; 95% CI: confidence interval; AOR: adjusted odds ratio; *P < 0.05; ^†^ *P* < 0.01; ^‡^ *P* < 0.001.

## References

[1] China CDC (2012). *National Behavioral and Biological Surveillance Report 2012*.

[2] Ministry of Health and Joint United Nations Programme on HIV/AIDS (2012). *2011 Estimates for the HIV/AIDS Epidemic in China*.

[3] van Griensven F, van Wijngaarden JWDL, Baral S, Grulich A (2009). The global epidemic of HIV infection among men who have sex with men. *Current Opinion in HIV and AIDS*.

[4] Wei C, Guadamuz TE, Stall R, Wong FY (2009). STD prevalence, risky sexual behaviors, and sex with women in a national sample of Chinese men who have sex with men. *American Journal of Public Health*.

[5] Zhang B, Li X, Shi T (2002). A preliminary estimate to population size of MSM, and HIV infection rate among MSM in China. *Chinese Journal of AIDS/STDs*.

[6] Grossman AH (1991). Gay men and HIV/AIDS: understanding the double stigma. *The Journal of the Association of Nurses in AIDS Care*.

[7] Li X, Lu H, Ma X (2012). HIV/AIDS-related stigmatizing and discriminatory attitudes and recent HIV testing among men who have sex with men in Beijing. *AIDS and Behavior*.

[8] Letamo G (2003). Prevalence of, and factors associated with, HIV/AIDS-related stigma and discriminatory attitudes in Botswana. *Journal of Health, Population and Nutrition*.

[9] Genberg BL, Hlavka Z, Konda KA (2009). A comparison of HIV/AIDS-related stigma in four countries: negative attitudes and perceived acts of discrimination towards people living with HIV/AIDS. *Social Science & Medicine*.

[10] Hao W, Su Z, Chen H (2007). Drinking and drinking-lelated problems in China. *Japanese Journal of Alcohol Studies & Drug Dependence*.

[11] Matthews AK, Cho YI, Hughes T, Wilsnack SC, Johnson T, Martin K (2013). The relationships of sexual identity, hazardous drinking, and drinking expectancies with risky sexual behaviors in a community sample of lesbian and bisexual women. *Journal of the American Psychiatric Nurses Association*.

[12] Cook RL, Clark DB (2005). Is there an association between alcohol consumption and sexually transmitted diseases? A systematic review. *Sexually Transmitted Diseases*.

[13] Genberg BL, Kawichai S, Chingono A (2008). Assessing HIV/AIDS stigma and discrimination in developing countries. *AIDS and Behavior*.

[14] Mahajan AP, Sayles JN, Patel VA (2008). Stigma in the HIV/AIDS epidemic: a review of the literature and recommendations for the way forward. *AIDS*.

[15] Ford K, Wirawan DN, Sumantera GM, Sawitri AAS, Stahre M (2004). Voluntary HIV testing, disclosure, and stigma among injection drug users in Bali, Indonesia. *AIDS Education and Prevention*.

[16] Rudolph AE, Davis WW, Quan VM (2012). Perceptions of community- and family-level injection drug user (IDU)- and HIV-related stigma, disclosure decisions and experiences with layered stigma among HIV-positive IDUs in Vietnam. *AIDS Care: Psychological and Socio-Medical Aspects of AIDS/HIV*.

[17] Kalichman SC, Simbayi LC, Kaufman M, Cain D, Jooste S (2007). Alcohol use and sexual risks for HIV/AIDS in sub-saharan Africa: systematic review of empirical findings. *Prevention Science*.

[18] Fisher JC, Bang H, Kapiga SH (2007). The association between HIV infection and alcohol use: a systematic review and meta-analysis of African studies. *Sexually Transmitted Diseases*.

[19] Steward WT, Miege P, Choi KH (2013). Charting a moral life: the influence of stigma and filial duties on marital decisions among Chinese men who have sex with men. *PLoS ONE*.

[20] Khan SI, Hudson-Rodd N, Saggers S, Bhuiya A (2005). Men who have sex with men’s sexual relations with women in Bangladesh. *Culture, Health and Sexuality*.

[21] Beichuan Z, Dianchang L, Xiufang L, Tiezhong H (2000). A survey of men who have sex with men: Mainland China. *American Journal of Public Health*.

[22] Choi K-H, Gibson DR, Han L, Guo Y (2004). High levels of unprotected sex with men and women among men who have sex with men: a potential bridge of HIV transmission in Beijing, China. *AIDS Education and Prevention*.

[23] Liao M, Kang D, Jiang B (2011). Bisexual behavior and infection with HIV and syphilis among men who have sex with men along the east coast of China. *AIDS Patient Care and STDs*.

[24] Folch C, Marks G, Esteve A, Zaragoza K, Muñoz R, Casabona J (2006). Factors associated with unprotected sexual intercourse with steady male, casual male, and female partners among men who have sex with men in Barcelona, Spain. *AIDS Education and Prevention*.

[25] Hernandez AL, Lindan CP, Mathur M (2006). Sexual behavior among men who have sex with women, men, and Hijras in Mumbai, India—multiple sexual risks. *AIDS and Behavior*.

[26] Lau JTF, Wang M, Wong HN (2008). Prevalence of bisexual behaviors among men who have sex with men (MSM) in China and associations between condom use in MSM and heterosexual behaviors. *Sexually Transmitted Diseases*.

[27] He Q, Wang Y, Lin P (2006). Potential bridges for HIV infection to men who have sex with men in Guangzhou, China. *AIDS and Behavior*.

[28] Bharat S (2011). A systematic review of HIV/AIDS-related stigma and discrimination in India: current understanding and future needs. *SAHARA-J: Journal of Social Aspects of HIV/AIDS*.

[29] Jiang J, Cao N, Zhang J (2006). High prevalence of sexually transmitted diseases among men who have sex with men in Jiangsu Province, China. *Sexually Transmitted Diseases*.

[30] Liu H, Li X, Stanton B (2005). Relation of sexual risks and prevention practices with individuals’ stigmatising beliefs towards HIV infected individuals: an exploratory study. *Sexually Transmitted Infections*.

[31] Ti L, Kerr T (2013). Task shifting redefined: removing social and structural barriers to improve delivery of HIV services for people who inject drugs. *Harm Reduction Journal*.

