# Supplementary material for: Alcohol Use, Stigmatizing/Discriminatory Attitudes, and HIV High-Risk Sexual Behaviors among Men Who Have Sex with Men in China
Source: Biomed Res Int. 2014 Mar 27;2014:143738. doi: 10.1155/2014/143738 (PMC3985170; doi:10.1155/2014/143738)
Supplement: Supplementary file 1 — Table 1. Demographics and Biological Outcomes among Men Who Have Sex with Men in Shandong Province, China. Table 2. Sex and Drug Use Behavior, Stigma and Discrimination, Alcohol Consumption, HIV Knowledge and HIV Prevention Services among Men Who Have Sex with Men in Shandong Province, China. Table 3. Predictors for Stigma and Discrimination, Alcohol Consumption among Men Who Have Sex with Men, Shandong Province, China. Figure 1. Comparisons of HIV/AIDS Related Stigmatizing and Discriminatory Attitudes with the Alcohol Consumption for the Participants Who Reported Ever Drinking≥3 Times per Week in Past 6 Months (P6M), Shandong Province, China. [file 143738.f1.doc]

**Table 1 Demographics and Biological Outcomes among Men Who Have Sex with Men in Shandong Province, China**

| **Variables** | **Total** | | **Drinking**  **(≥3 times/week)** | | **Stigma and Discrimination** |
| --- | --- | --- | --- | --- | --- |
|  | **N** | **%** | **N** | **%** | **±SD** |
| Total | 1230 |  | 144 | 11.7 | 37.4±4.4 |
| **Demographics** |  |  |  |  |  |
| Study sites |  |  |  |  |  |
| Jinan | 400 | 32.5 | 48 | 12.0 | 39.0±3.8 |
| Qingdao | 400 | 32.5 | 55 | 13.7 | 33.8±3.8‡ |
| Yantai | 430 | 35.0 | 41 | 9.5 | 39.4±2.9 |
| Recruited venue |  |  |  |  |  |
| Bars, night clubs, or tea houses | 329 | 26.7 | 49 | 14.9 | 36.7±4.2 |
| Bathhouses or sauna | 161 | 13.1 | 24 | 14.9 | 33.8±3.8‡ |
| Outdoor cruising area | 98 | 8.0 | 11 | 11.2 | 34.1±3.9‡ |
| Internet or HIV testing sites | 642 | 52.2 | 60 | 9.3 | 39.2±3.6 |
| Age (years) |  |  |  |  |  |
| <25 | 548 | 44.6 | 53 | 9.7 | 37.8±4.2 |
| 25-34 | 505 | 41.1 | 50 | 9.9 | 37.4±4.4 |
| ≥35 | 177 | 14.3 | 41 | 23.2‡ | 36.1±4.6‡ |
| Marital status |  |  |  |  |  |
| Single/separated | 1018 | 82.8 | 97 | 9.5 | 37.7±4.2 |
| Married or cohabitating | 212 | 17.2 | 47 | 22.2‡ | 35.9±4.6‡ |
| Residency |  |  |  |  |  |
| Shandong Province | 991 | 80.6 | 107 | 10.8 | 37.4±4.4 |
| Non-Shandong Province | 239 | 19.4 | 37 | 15.5 | 37.6±4.2 |
| Ethnicity group |  |  |  |  |  |
| Han | 1206 | 98.0 | 140 | 11.6 | 37.4±4.4 |
| Others | 24 | 2.0 | 4 | 16.7 | 38.8±3.5 |
| Occupation |  |  |  |  |  |
| Student | 187 | 15.2 | 6 | 3.2 | 38.3±4.3 |
| Commercial service | 539 | 43.8 | 80 | 14.8 | 37.7±4.2 |
| Farmer | 117 | 9.5 | 8 | 6.8 | 38.9±4.0 |
| Full time employee | 268 | 21.8 | 33 | 22.9‡ | 37.0±4.3 |
| Housework and/or unemployed | 119 | 9.7 | 17 | 14.3 | 34.6±4.3‡ |
| Education |  |  |  |  |  |
| High school or lower | 649 | 52.8 | 94 | 14.5† | 36.9±4.5‡ |
| College or higher | 581 | 47.2 | 50 | 8.6 | 38.0±4.1 |
| Duration of residence in current location (years) |  |  |  |  |  |
| ≥2 | 815 | 66.3 | 97 | 11.9 | 37.4±4.4 |
| <2 | 415 | 33.7 | 47 | 11.3 | 37.4±4.2 |
| Self-identified sexual orientation |  |  |  |  |  |
| Homosexual | 845 | 68.7 | 73 | 8.6 | 37.6±4.2 |
| Heterosexual | 17 | 1.4 | 6 | 35.3‡ | 34.6±4.7‡ |
| Bisexual | 323 | 26.3 | 61 | 18.9 | 36.9±4.6 |
| Do not know | 45 | 3.7 | 4 | 8.9 | 39.2±3.1 |
| Being married or cohabitating/ever had sex with woman in past 6 months |  |  |  |  |  |
| Homosexual | 878 | 71.4 | 74 | 8.4 | 37.8±4.3 |
| Bisexual | 352 | 28.6 | 70 | 19.9‡ | 36.6±4.5‡ |
| **Biological outcome** |  |  |  |  |  |
| HIV status |  |  |  |  |  |
| Negative | 1208 | 98.4 | 142 | 11.8 | 37.4±4.3 |
| Positive | 20 | 1.6 | 2 | 10.0 | 38.3±4.4 |
| Syphilis status |  |  |  |  |  |
| Negative | 1144 | 93.2 | 130 | 11.4 | 37.5±4.4 |
| Positive | 84 | 6.8 | 14 | 16.7 | 36.4±4.2* |

Note: Total N for each subgroup may not add up to the total due to missing data; P6M: in the past 6 months; ^*^: P<0.05; †: p<0.01; ‡: P<0.001; NA: not applicable

**Table 2 Sex and Drug Use Behavior, Stigma and Discrimination, Alcohol Consumption, HIV Knowledge and HIV Prevention Services among Men Who Have Sex with Men in Shandong Province, China**

| **Variables** | **Total** | | **Drinking**  **(≥3 times/week)** | | **Stigma and Discrimination** |
| --- | --- | --- | --- | --- | --- |
|  | **N** | **%** | **N** | **%** | **±SD** |
| **Sexual and drug use behaviors** |  |  |  |  |  |
| Age of first sex (years) |  |  |  |  |  |
| ≤20 | 661 | 53.7 | 70 | 10.6 | 38.0±4.1 |
| >20 | 569 | 46.3 | 74 | 13.0 | 36.7±4.6‡ |
| Sex with man in past 6 months |  |  |  |  |  |
| No | 105 | 8.6 | 6 | 5.7 | 39.4±3.2 |
| Yes | 1123 | 91.4 | 138 | 12.3 | 37.2±4.4‡ |
| No. of male sex partners in the past week |  |  |  |  |  |
| <2 | 499 | 45.7 | 43 | 8.6 | 38.8±3.9 |
| ≥2 | 592 | 54.3 | 92 | 15.5† | 35.9±4.4‡ |
| Condom use during sex with man in the last sex |  |  |  |  |  |
| Yes | 794 | 70.8 | 89 | 11.2 | 37.6±4.3 |
| No | 327 | 29.2 | 48 | 14.7 | 36.4±4.5‡ |
| Condom use during sex with man in past 6 months |  |  |  |  |  |
| Always | 351 | 31.3 | 27 | 7.7 | 39.1±3.4 |
| Sometimes or never | 770 | 68.7 | 111 | 14.4† | 36.4±4.5‡ |
| Commercial sex with man in past 6 months |  |  |  |  |  |
| Yes | 306 | 27.2 | 51 | 16.7† | 35.7±4.2‡ |
| No | 818 | 72.8 | 86 | 10.5 | 37.8±4.3 |
| Condom use with paid male partner during the last sex |  |  |  |  |  |
| No | 84 | 6.8 | 18 | 21.4 | 34.3±4.4‡ |
| Yes | 222 | 72.5 | 33 | 14.9 | 36.2±4.0 |
| Condom use with paid male sex partners in past 6 months |  |  |  |  |  |
| Always | 91 | 29.8 | 12 | 13.2 | 38.4±3.4 |
| Sometimes or never | 214 | 70.2 | 38 | 17.8 | 34.5±4.0‡ |
| Sold sex to man in past 6 months |  |  |  |  |  |
| Yes | 264 | 21.5 | 43 | 16.3^*^ | 36.1±4.2‡ |
| No | 966 | 78.5 | 101 | 10.5 | 37.8±4.3 |
| Condom use with sold male sex partner the last time |  |  |  |  |  |
| No | 56 | 21.4 | 9 | 16.1 | 34.8±4.3‡ |
| Yes | 206 | 78.6 | 34 | 16.5 | 36.4±4.1 |
| Condom use with sold male sex partners in past 6 months |  |  |  |  |  |
| Always | 82 | 31.1 | 12 | 14.6 | 38.8±3.2 |
| Sometimes or never | 182 | 68.9 | 31 | 17.0 | 34.9±4.1‡ |
| Sex with woman in past 6 months |  |  |  |  |  |
| Yes | 287 | 23.4 | 61 | 21.3‡ | 36.8±4.5† |
| No | 942 | 76.6 | 83 | 8.8 | 37.6±4.3 |
| Condom use with female partners in the last sex act |  |  |  |  |  |
| Yes | 149 | 52.1 | 23 | 15.4 | 37.7±4.1 |
| No | 137 | 47.9 | 37 | 27.0^*^ | 35.9±4.7‡ |
| Condom use with female partners in past 6 months |  |  |  |  |  |
| Always | 93 | 32.5 | 14 | 15.1 | 38.4±3.7 |
| Sometimes or never | 193 | 67.5 | 46 | 23.8 | 36.1±4.6‡ |
| Drug use |  |  |  |  |  |
| No | 1210 | 98.9 | 138 | 11.4 | 37.4±4.4 |
| Yes | 13 | 1.1 | 6 | 46.2† | 36.9±3.8 |
| **HIV-related prevention services in the past year** |  |  |  |  |  |
| Condom promotion/VCT |  |  |  |  |  |
| Yes | 925 | 75.2 | 116 | 12.5 | 38.1±4.1 |
| No | 305 | 24.8 | 28 | 9.2 | 37.2±4.4† |
| Received peer education |  |  |  |  |  |
| Yes | 508 | 41.3 | 69 | 13.6 | 38.7±4.0 |
| No | 722 | 58.7 | 75 | 10.4 | 36.5±4.3‡ |
| Had free HIV test in the past year |  |  |  |  |  |
| Yes | 624 | 50.7 | 87 | 13.9^*^ | 38.5±3.9 |
| No | 606 | 49.3 | 57 | 9.4 | 36.3±4.5‡ |
| **Drink ≥3 times per week in P6M** |  |  |  |  |  |
| No | 1086 | 88.3 | - | - | 37.6±4.2 |
| Yes | 144 | 11.7 | - | - | 35.4±5.2‡ |

Note: Total N for each subgroup may not add up to the total due to missing data; P6M: in the past 6 months; ^*^: P<0.05; †: p<0.01; ‡: P<0.001; NA: not applicable

**Table 3 Predictors for Stigma and Discrimination, Alcohol Consumption among Men Who Have Sex with Men, Shandong Province, China.**

| **Model 1 Predictors for Drink ( ≥3 times per week in P6M)** | **N (%)** | **OR (95%CI)** | **AOR (95%CI)** |
| --- | --- | --- | --- |
| Higher level of stigma and discrimination (continuous) | 35.4±5.2 | 0.90(0.86-0.93)‡ | 0.92(0.87-0.96)‡ |
| Unprotected male anal sex in P6M | 111(14.4) | 2.0(1.3-3.1)† | 1.9(1.1-3.3)^*^ |
| Bisexual identity/orientation | 70(19.9) | 2.7(1.9-3.8)‡ | 2.2(1.3-3.9)† |
| No. of male sex partners in the past week ≥2 | 92(15.5) | 2.0(1.3-2.9)† | 1.5(1.0-2.4)^*^ |
| Drug use | 6(46.2) | 6.7(2.2-20.1)† | 6.7(2.0-22.3)† |
| High school or lower education level | 94(14.5) | 1.7(1.1-2.5)† | 1.6(1.0-2.6)^*^ |
| **Model 2 Predictors for stigma and discrimination** | **Mean**±**SD** | **β(95%CI)** | **Adjustedβ(95%CI)** |
| Drink ≥3 times per week in P6M | 35.4±5.2 | 2.2(1.5-3.0)‡ | 1.5(0.8-2.3)‡ |
| Unprotected male anal sex in P6M | 36.4±4.5 | 2.8 (2.2-3.3)‡ | 1.7(1.1-2.2)‡ |
| Bisexual identity/orientation | 36.6±4.5 | 1.2 (0.6-1.7)‡ | 0.9 (0.4-1.4)† |
| No. of male sex partners in past week ≥2 | 35.9±4.4 | 2.9 (2.4-3.4)‡ | 1.9 (1.3-2.4)‡ |
| Commercial sex with man in P6M | 35.7±4.2 | 2.1(1.5-2.6)‡ | 0.8(0.2-1.4)† |
| Never received peer education in the past year | 36.5±4.3 | 2.3 (1.8-2.7)‡ | 1.7 (1.2-2.2)‡ |

Note: Multivariable logistic regression analysis was applied for alcohol consumption (Model 1); Multivariable linear regression model was performed for stigma and discrimination (Model 2); P6M: in the past 6 months; OR: odds ratio; 95%CI: confidence interval; AOR: adjusted odds ratio; ^*^: P<0.05; †: p<0.01; ‡: P<0.001.

**FIGURE LEGEND**

**Figure 1 Comparisons of HIV/AIDS Related Stigmatizing and Discriminatory Attitudes with the Alcohol Consumption for the Participants Who Reported Ever Drinking≥3 Times per Week in Past 6 Months (P6M), Shandong Province, China.**


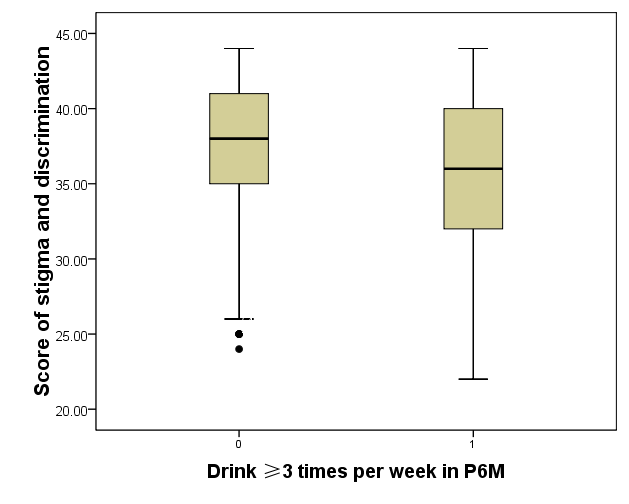


Yes

P<0.001

No
